# Subintimal intravascular lithotripsy to optimize external crush stenting

**DOI:** 10.1093/ehjcr/ytae229

**Published:** 2024-04-25

**Authors:** Alexandru Achim, Zoltan Jambrik, Ferenc Nagy, Zoltan Ruzsa

**Affiliations:** Department of Internal Medicine, Invasive Cardiology Division, University of Szeged, Semmelweis u. 8, Szeged 6725, Hungary; Department of Cardiology, ‘Niculae Stancioiu’ Heart Institute, University of Medicine and Pharmacy ‘Iuliu Hatieganu’, Motilor 19-21, 400001 Cluj-Napoca, Romania; Department of Internal Medicine, Invasive Cardiology Division, University of Szeged, Semmelweis u. 8, Szeged 6725, Hungary; Department of Internal Medicine, Invasive Cardiology Division, University of Szeged, Semmelweis u. 8, Szeged 6725, Hungary; Department of Internal Medicine, Invasive Cardiology Division, University of Szeged, Semmelweis u. 8, Szeged 6725, Hungary

A 72-year-old male patient with a medical history marked by coronary artery disease and multiple triple-vessel stenting presented to our department due to pronounced angina and dyspnoea during minimal exertion, stemming from a long in-stent right coronary artery chronic total occlusion (CTO).

Employing an antegrade wire escalation technique, the wire could be advanced in the true lumen of the postero-descending artery, but with a long subintimal tracking, behind the old stent, as shown by intravascular ultrasound (IVUS). Contrary to prevailing beliefs advocating intentional subintimal tracking as a way to evade calcification,^1^ the new stent (parallel to the old one) could not expand or post-dilate at high pressures (*Figure 1A* and *B*; Supplementary material online, *Video S1*). This can be attributed to a dual challenge: the original stent, now in place for 5 years, had stiffened and accrued calcific neoatherosclerosis. Furthermore, IVUS revealed substantial extraplaque calcification, positioned opposite the initial stent (*Figure 1C*).

**Figure 1:**
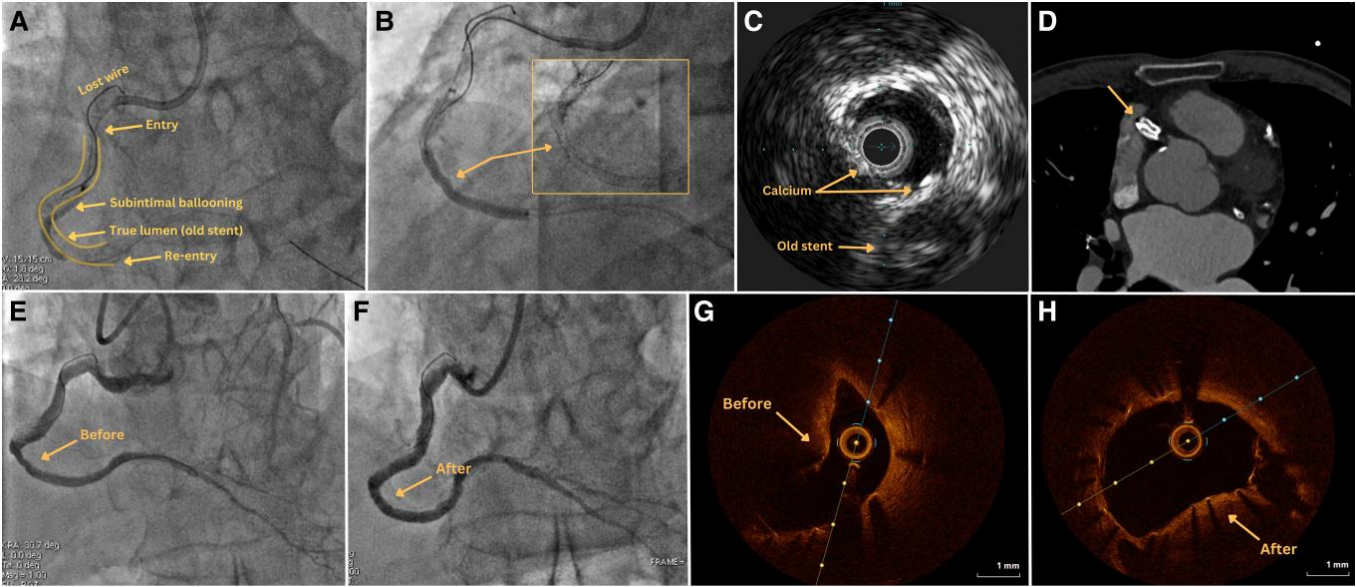
(*A*) Long, subintimal tracking outside the old stent during antegrade wiring. (*B*) Stent under-expansion in the subintimal segment (arrows). (*C*) Intra-vascular ultrasound confirms the intra-stent calcified chronic total occlusion and calcifications in the media. (*D*) ‘Double-barrel’ appearance of the two stents, computed tomography (arrow shows two parallel stents, one occluded situated in the ‘old true lumen’ and one subintimal, now realizing the permeable lumen). (*E*, *F*) Before and after intravascular lithotripsy of the subintimally located stent. (*G*, *H*) Optical coherence tomography before and after intravascular lithotripsy.

The patient was referred 3 months later for intravascular lithotripsy (IVL) at this level. Eighty pulsations were administered using a 3.5 mm balloon. The optical coherence tomography showed a well-endothelialized vessel, but with segments of under-expanded stent caused by a protruding calcified plaque [minimal stent area (MSA) 5.3 mm2]. Being a large vessel, the interrogation could not capture the depth of the old stent, but the computed tomography offered an evocative ‘double-barrel’ image (*Figure 1D*). Post-IVL and subsequent non-compliant 3.5 mm balloon dilation, an MSA of 10.1 mm^2^ was achieved (50% increase) (*Figure 1E–H*; Supplementary material online, *Video S1*). Finally, a 3.5 mm drug-eluting balloon (DEB) was applied.

In the context of in-stent CTO percutaneous coronary intervention (PCI) within heavily calcified vessels, subintimal IVL can modify the extraplaque calcium and improve vessel compliance to facilitate external crush stenting.^2^ This report not only re-affirms the safety of subintimal IVL post-endothelialization but also underscores the importance of incorporating plaque modification technologies in navigating through challenging calcified lesions, potentially superseding aggressive subintimal dilation methods. While the use of coronary IVL in the extraplaque space has been previously reported,^2^ it is still relatively rare due to the uncommon occurrence of significant extraplaque calcification. Given the potential risk of perforation associated with *ad hoc* extraplaque IVL,^3^ adopting a methodical stepwise strategy may be a more prudent approach, particularly when allowing the stent to undergo endothelialization. Given that the polymer of the stent remains unaffected by IVL shocks,^4^ but complete drug release occurs within 1–3 months, the recommendation is to incorporate a DEB at this stage. Chronic total occlusion PCI expertise remains essential when navigating such devices within the subintimal space.

## Supplementary Material

ytae229_Supplementary_Data

## Data Availability

The data underlying this article will be shared on reasonable request to the corresponding author.

## Supplementary material

**Consent:** Informed consent for patient information and images to be published was provided by the patients in accordance with COPE guidelines.

**Ethics approval:** This material is the authors’ own original work, which has not been previously published elsewhere.

**Funding:** None declared.
